# A Survey of Crop Disease Recognition Methods Based on Spectral and RGB Images

**DOI:** 10.3390/jimaging12020066

**Published:** 2026-02-05

**Authors:** Haoze Zheng, Heran Wang, Hualong Dong, Yurong Qian

**Affiliations:** 1 School of Computer Science and Technology, Xinjiang University, Urumqi 830046, China; zhenghaoze@stu.xju.edu.cn (H.Z.); 20231405209@stu.xju.edu.cn (H.W.); 107552301674@stu.xju.edu.cn (H.D.); 2 Joint International Research Laboratory of Silk Road Multilingual Cognitive Computing, Xinjiang University, Urumqi 830046, China; 3 Key Laboratory of Software Engineering, Xinjiang University, Urumqi 830091, China; 4 Xinjiang Engineering Research Center of Big Data and Intelligent Software, School of Software, Xinjiang University, Urumqi 830046, China

**Keywords:** crop disease recognition, spectral images, red–green–blue images, traditional machine learning, deep learning

## Abstract

Major crops worldwide are affected by various diseases yearly, leading to crop losses in different regions. The primary methods for addressing crop disease losses include manual inspection and chemical control. However, traditional manual inspection methods are time-consuming, labor-intensive, and require specialized knowledge. The preemptive use of chemicals also poses a risk of soil pollution, which may cause irreversible damage. With the advancement of computer hardware, photographic technology, and artificial intelligence, crop disease recognition methods based on spectral and red–green–blue (RGB) images not only recognize diseases without damaging the crops but also offer high accuracy and speed of recognition, essentially solving the problems associated with manual inspection and chemical control. This paper summarizes the research on disease recognition methods based on spectral and RGB images, with the literature spanning from 2020 through early 2025. Unlike previous surveys, this paper reviews recent advances involving emerging paradigms such as State Space Models (e.g., Mamba) and Generative AI in the context of crop disease recognition. In addition, it introduces public datasets and commonly used evaluation metrics for crop disease identification. Finally, the paper discusses potential issues and solutions encountered during research, including the use of diffusion models for data augmentation. Hopefully, this survey will help readers understand the current methods and effectiveness of crop disease detection, inspiring the development of more effective methods to assist farmers in identifying crop diseases.

## 1. Introduction

### 1.1. Overview

Agriculture is the cornerstone of national development and is crucial to the national economy. Currently, global food supplies are under strain, and crops, as a strategic and foundational core industry, often face significant impacts on their yield and quality due to climatic conditions, pests, and various pathogens [1]. Agriculture 4.0 has become a transformative trend in modern agriculture worldwide. Leveraging opportunities from the Internet of Things, machine learning, drones, big data analytics, and artificial intelligence can benefit all stages and processes of the agricultural production chain [2]. Tasks involved include disease recognition, yield estimation, and production management.

**Figure 1:**
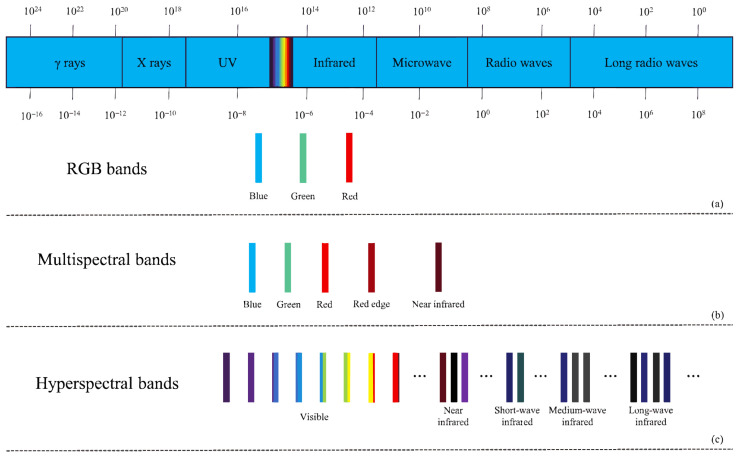
(**a**) RGB bands, (**b**) multispectral bands, (**c**) hyperspectral bands.

With the advancement of computer hardware, photography techniques, and computer technology, computer vision image recognition methods based on spectral and RGB images have attracted increasing attention from researchers. Figure 1 displays the band information of RGB images, multispectral images, and hyperspectral images. These methods find wide applications in agriculture, such as weed detection [3], crop yield estimation [4], and more. They alleviate the subjectivity, workload, and time constraints associated with manual recognition and significantly enhance the efficiency and accuracy of related tasks.

Thousands of common crop diseases are identified [5], severely impacting global crop yields and resulting in significant economic losses annually. Therefore, it is crucial to prevent and control crop diseases through disease recognition methods. However, traditional disease recognition typically relies on experts visually inspecting plant disease symptoms using microscopes or conducting physicochemical analyses, requiring significant human and material resources and specialized knowledge and equipment. This approach fails to meet the accuracy and flexibility requirements of modern agriculture [6]. Moreover, lacking specialized knowledge, farmers often use large quantities of chemicals to prevent crop diseases, which can effectively suppress disease occurrence but may damage soil environments and pose numerous health risks [7].

In order to tackle the challenges above effectively, many researchers are integrating spectral and RGB images associated with crop diseases with artificial intelligence technologies. It has increasingly made disease recognition methods based on spectral and RGB images a critical safeguard for crop yields, food security, and the health of animals and humans [8], elevating its significance in addressing real-world agricultural issues. The ongoing enhancement and improvement of crop disease recognition methods not only aid in the timely detection of crop diseases, the optimization of agricultural practices, and the prompt development of disease eradication strategies but also support farmers who may lack specialized knowledge in minimizing the use of chemicals, ensuring the sustainable growth of crop farming.

### 1.2. Methodology and Contributions

This paper reviews the existing crop disease recognition methods based on spectral and RGB images. To ensure the comprehensiveness and reliability of the content, we initially selected articles from various academic resources such as Google Scholar, ELSEVIER, Scopus, Frontiers, IEEE Xplore, and Springer using keywords such as “plant/crop disease detection/recognition/classification”, “spectral image-based disease recognition”, and “RGB image-based disease recognition”. Articles from 2020 to 2025 were screened based on their relevance to this survey, the significance of the methods employed, the performance of the models, and the datasets used.

There are also several literature reviews related to crop disease identification technology.

For instance, Iqbal et al. [9] proposed a detailed classification system for citrus diseases, encompassing preprocessing, segmentation, feature extraction, feature selection, and classification, along with their challenges, advantages, and disadvantages. Additionally, it delineated the gaps between methods introduced to enhance detection and classification accuracy. More recently, Upadhyay et al. [10] provided a comprehensive review of deep learning techniques in precision agriculture published in 2025, highlighting the paradigm shift towards real-time detection models, Generative AI, and Foundation Models, which complements the scope of earlier reviews. The review study by Noon et al. [11] focused on the performance of Convolutional Neural Network (CNN) models in detecting diseases in vegetables, fruits, and miscellaneous plant species. Abade et al. [12] primarily reviewed the techniques of CNN in crop disease identification and classification, investigating significant contributions to the listed challenges and different innovations to improve CNN performance. They also summarized available methods based on the models, datasets, and types of CNNs used for plant disease detection. Terentev et al. [13] analyzed the current state of early plant disease detection using hyperspectral remote sensing in four different crops: oil palm, citrus, solanaceae, and wheat. They demonstrated the feasibility of early plant disease detection through hyperspectral remote sensing. They proposed a systematic table for detecting plant diseases via hyperspectral remote sensing, including critical wavelength bands and sensor model information. Thakur et al. [14] briefly investigated the types of equipment used in data capture, capture conditions, regions for dataset collection, and preprocessing techniques. Furthermore, they introduced various public datasets used by researchers in this field. The article then discussed the impact of emerging trends in deep learning, such as transfer learning, attention mechanisms, localization methods, and lightweight models in plant disease identification. It examined the suitability of attention-based convolutional neural networks for field plant disease identification. Finally, the article elaborated on the plant diseases considered necessary in different countries. Jafar et al. [15] emphasized the most common diseases and infections in four vegetables: tomato, pepper, potato, and cucumber, along with their symptoms. They provided detailed predetermined steps for predicting plant diseases using artificial intelligence, including image acquisition, preprocessing, segmentation, feature selection, and classification. They also comprehensively reviewed machine learning-based and deep learning-based research to detect diseases in these four crops, including datasets used to evaluate these studies.

Despite the existing review studies that have covered various methods and pointed out significant challenges, there is still a need to revisit the approaches for crop disease identification from different perspectives to assess the strengths and weaknesses of the current methods. The foundation of disease identification research lies in data, and training models capable of accurately identifying diseases requires the assembly of credible datasets comprising various disease images. Therefore, unlike previous reviews, this paper focuses on the types of images frequently used for crop disease identification. Firstly, it reviews the available spectral and RGB public datasets for crop disease identification and the evaluation metrics used for assessing crop disease identification models, helping readers better understand model performance and conduct related experiments. Subsequently, it summarizes the crop disease identification techniques based on spectral and RGB images and discusses some limitations of these techniques in crop disease identification, along with possible improvement strategies. Additionally, the paper discusses the differences among different crop disease identification methods and the considerations for researching disease identification. Finally, the paper summarizes potential future research directions for disease identification based on spectral and RGB images, including the combination of spectral and RGB and the generation of disease images using diffusion techniques. Table 1 summarizes the coverage and objectives of the existing review studies and the review study proposed in this paper.

The remaining content of this review is as follows: Section 2 summarizes the public datasets and model performance evaluation metrics used for crop disease identification. Section 3 introduces methods for crop disease identification based on spectral images, mainly summarizing the commonly used traditional machine learning methods for disease identification based on spectral images, as well as the limitations of these methods and possible improvement strategies. Section 4 introduces methods for crop disease identification based on RGB images, mainly summarizing the commonly used deep learning methods for disease identification based on RGB images, as well as the limitations of these methods and possible improvement strategies. Section 5 discusses the precautions for different crop disease identification methods. Section 6 summarizes and outlines the future directions of crop disease identification technologies.

## 2. Public Datasets and Evaluation Metrics

### 2.1. Public Datasets

**Figure 2:**
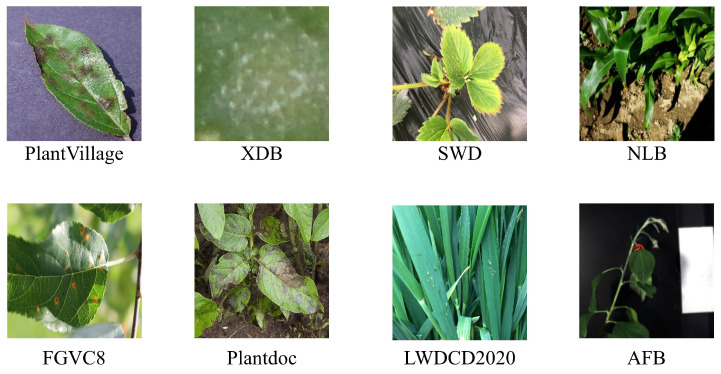
Sample images from various datasets.

When developing efficient plant disease recognition methods, the availability of sufficient high-quality datasets is crucial. Some researchers have provided public datasets covering a wide range of plant diseases. Table 2 summarizes the publicly available spectral and RGB image datasets for disease recognition. These datasets include various disease types and can be used to train different disease recognition models. Figure 2 shows some examples from these datasets.

1. PlantVillage—The PlantVillage dataset is widely used for crop disease classification tasks. It covers 38 crop disease categories across 14 plant species, with 54,305 images [16]. All images were obtained in controlled laboratory environments with simple backgrounds.
2. XDB [17] from Embrapa Agricultural Research Institute introduced the Plant Disease Detection Database (PDDB), covering 171 diseases and other conditions across 21 plant species, with 2326 images as of October 2016. To support powerful technologies like deep learning, this dataset was expanded into the XDB dataset, where each image was subdivided according to specific criteria, increasing the total number of images to 46,513. However, as indicated in Table 2, most public datasets are collected in controlled environments. To address the scarcity of diverse field data, recent studies have increasingly explored generative models for data augmentation. Han et al. [26] demonstrated that GAN-based synthesis can significantly improve disease detection robustness. Moreover, recent reviews highlight diffusion-based augmentation as a promising direction for enhancing generalization in real-world agricultural scenarios [27].
3. SWD [18] constructed a large dataset of 3531 images to evaluate and test their proposed method. The dataset includes four categories: Healthy leaf, Healthy petiole, Verticilium leaf, and Verticilium petiole. Each image indicates whether the strawberry plant is infected with Verticillium wilt disease.
4. NLB—NLB is an object detection dataset created by [19] specifically for northern corn leaf blight. It consists of 18,222 images collected from actual fields using smartphones, fixed cameras, and drones, with annotations of 105,735 bounding boxes for northern corn leaf blight lesions.
5. FGVC8 [20] manually captured 3651 high-quality images of actual symptoms, showcasing various apple leaf diseases with different lighting, angles, surfaces, and noise. Based on this, they created pilot datasets for apple scab, cedar rust, and healthy leaves provided to the Kaggle community and named FGVC7 for the Fine-Grained Visual Categorization Challenge at CVPR2020. FGVC8 significantly increased the number of apple leaf disease images from FGVC7 and added more segmented disease categories, totaling 18,632 images covering 12 categories.
6. LWDCD2020—LWDCD2020 comprises approximately 12,000 images covering nine wheat disease categories (loose smut, spot blotch, powdery mildew, leaf rust, Fusarium head blight, crown rot, black point, Karnal bunt, and wheat streak mosaic virus) and one healthy category [21]. These images have undergone dimensionally unified preprocessing. Almost all images in LWDCD2020 contain only one type of disease and exhibit complex backgrounds, varying acquisition conditions, features from different disease development stages, and similar features among different wheat diseases.
7. Plantdoc—The Plantdoc dataset consists of 2598 images covering 13 plant species and 17 disease categories [22]. It is a publicly available dataset used for object detection.
8. BARI-Sunflower—The BARI-Sunflower dataset was constructed from collections at the Bangladesh Agricultural Research Institute (BARI) Gazipur Demonstration Farm, comprising 467 original images of healthy and diseased sunflower leaves and flowers [23]. To meet the demands of deep learning for data, data augmentation techniques such as random rotation, scaling, and cropping were applied, resulting in 470 images of downy mildew, 509 images of leaf scars, 398 images of gray mold, and 515 images of fresh leaves.
9. Katra-Twelve—Katra-Twelve is a public leaf image dataset provided by Shri Mata Vaishno Devi University, encompassing images of healthy and diseased leaves [24]. The dataset features 12 economically and environmentally beneficial plant species, covering 22 leaf types. There are 4503 images, with 2278 images of healthy leaves and 2225 images of diseased leaves.
10. AFB—The dataset by [25] comprises three hyperspectral images of apple tree plants. The first set of images involves a 15-day monitoring period of seven apple trees infected with fire blight and six control plants. The second set of images includes time monitoring of three infected plants, seven plants subjected to water stress, and seven control plants. The third set of images was collected in an orchard, involving nine trees exhibiting symptoms of fire blight and six control trees. All images displaying symptomatic plants provide pixel location information for the infected areas.

### 2.2. Evaluation Metrics

In recent years, multiple evaluation metrics have been introduced to assess the performance of disease identification models. Standard metrics for evaluating machine learning disease identification models based on spectral images include accuracy, precision, Matthews correlation coefficient (MCC), and kappa coefficient. Commonly used metrics for disease detection models based on RGB images include average precision (AP), mean average precision (mAP), F1 score, accuracy, and recall. Additionally, some studies estimate the severity of disease occurrence while identifying crop diseases. There are evaluation metrics for assessing the accuracy of these estimates, such as root mean square error (RMSE), R-squared ($R^{2}$), and mean absolute error (MAE). This section summarizes evaluation metrics for assessing disease model recognition performance and evaluating the accuracy of estimating disease severity in Table 3.

## 3. Crop Disease Recognition Methods Based on Spectral Images

Spectral imaging technology originated from multispectral remote sensing technology in the 1970s, which typically includes 3 to 10 discrete bands such as red, green, and blue, primarily covering specific bands within the visible and near-infrared spectral ranges but failing to provide continuous spectral information. Spectral imaging technology has continually evolved with the need for Earth remote sensing, leading researchers to develop further hyperspectral sensors capable of capturing tens or even hundreds of continuous narrow bands from the visible to infrared spectral ranges, assigning each pixel of the spatial image with its unique spectral information. Figure 1 illustrates the band information of these two types of spectral images. Thanks to the characteristics of spectral images, researchers can analyze the chemical composition and inherent physical structure differences of crops, obtaining information that reflects the biological status of crops, similar to the Normalized Difference Vegetation Index (NDVI) [28]. Abbott et al. [29] was the first to apply spectral imaging to the agricultural field, achieving quality measurement of fruits and vegetables. Judith’s success attracted many researchers to apply spectral imaging to agriculture, gradually becoming one of the most commonly used image types for crop disease identification. Most existing research on disease detection based on spectral images has been conducted using machine learning algorithms and has achieved excellent results. While traditional machine learning methods have long dominated spectral disease analysis, recent studies have increasingly applied deep learning architectures to hyperspectral data. Goyal et al. [30] employed hybrid deep learning models to extract spectral-spatial features for early disease detection. Similarly, Zhang et al. [31] utilized 3D-CNNs to improve classification accuracy in apple quarantine diseases. Furthermore, Chossegros et al. [32] demonstrated the effectiveness of deep learning in distinguishing multiple overlapping infections in wheat.

### 3.1. Spectral Image-Based Traditional Machine Learning Crop Disease Recognition Methods

**Figure 3:**
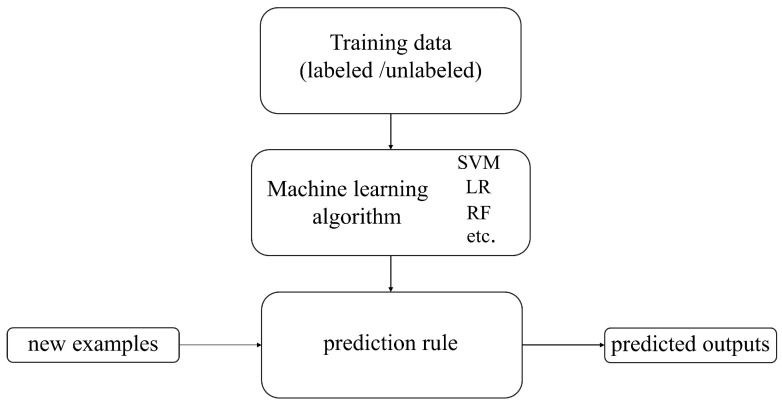
The basic process of machine learning algorithms.

Machine learning is a branch of computer science that aims to enable computers to “learn” without direct programming [33]. Figure 3 shows the basic process of machine learning algorithms. The development history of traditional machine learning is a continuously evolving and expanding process. Since the 1950s, machine learning, as an essential branch of artificial intelligence, has begun to receive attention and has transitioned from early research to the exploration of knowledge implantation and symbolic representation and then to the revitalization and multi-concept learning stage. Machine learning technology has continuously matured and gradually integrated with multiple disciplines, such as psychology, biology, mathematics, automation, and computer science, forming a solid theoretical foundation. With the continuous advancement of technology and the increasing demand for applications, machine learning has been widely applied since the mid-1980s, covering various fields such as finance, e-commerce, transportation, entertainment, and manufacturing. These applications have driven the development of related industries and further promoted the improvement and innovation of machine learning technology. Today, machine learning is widely applied in the agricultural field. Researchers provide rich suggestions and insights about crops to help machines learn crop characteristics, assisting farmers in reducing agricultural losses, achieving more efficient and precise agricultural management, and improving production quality [34].

**Figure 4:**
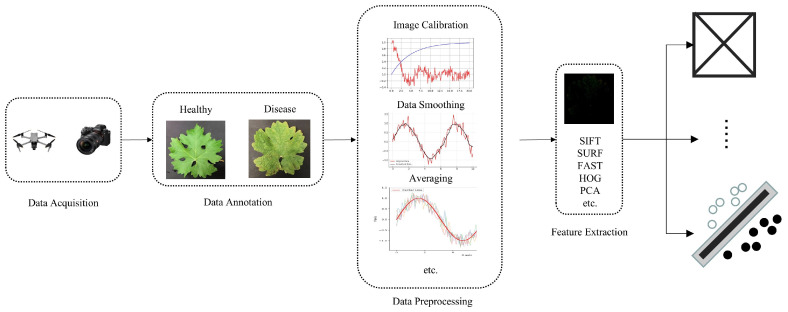
The basic process of spectral image-based machine learning disease recognition methods.

Since spectral images are correlated with the biophysical and biochemical properties of crops, they can assist researchers in analyzing the chemical composition and inherent physical structure differences of crops, thereby achieving the purpose of monitoring crop health status. Traditional machine learning methods primarily utilize the spectral information of images when processing spectral image data, placing greater emphasis on the spectral characteristics of each pixel, which can improve the accuracy of crop disease identification. Therefore, an increasing number of researchers are utilizing various traditional machine learning methods to develop crop disease classification methods based on spectral images. Figure 4 illustrates the basic process of machine learning-based disease identification methods using spectral images.

Based on the selection criteria of this paper, 27 studies on spectral image-based machine-learning disease recognition methods from 2020 to 2024 were selected. Table 4 shows the studies on spectral image-based machine learning disease recognition methods chosen for this paper, including publication time, references, recognition disease, camera model, wavelength, machine learning algorithms (highlighted in bold for the best-performing ones), and the optimal recognition results.

### 3.2. Limitations of Spectral Image-Based Traditional Machine Learning Crop Disease Recognition Methods

From Table 4, we can observe that machine learning algorithms such as SVM, Random Forest (RF), and K-Nearest Neighbors (KNN) can effectively utilize spectral imagery for crop disease classification and severity prediction. However, these input-intensive methods currently face several limitations regarding theory, technology, and practical applications.

#### 3.2.1. Limitations of Data Processing and Traditional Machine Learning Methods

From Figure 4, we can observe that when using traditional machine learning methods for crop disease identification, data preprocessing is required to correct or restore spectral images in order to eliminate data anomalies caused by noise from factors such as geometric shape and atmospheric condition changes [62], as well as geometric parameters of sensors and plants. This preprocessing step facilitates the extraction of useful information. However, different types of spectral images, wavebands, or capture devices may necessitate different preprocessing techniques, posing challenges in data processing. Following data preprocessing, steps such as designing feature extractors by experienced experts are necessary to convert the preprocessed crop disease data into intermediate representations or feature vectors rich in disease characteristics. These features are then used for classification by traditional machine learning algorithms.

Although manual feature extraction has achieved good results so far, it is subject to issues such as strong subjectivity and inefficiency, and cannot achieve end-to-end applications. Therefore, establishing standard data preprocessing and feature extraction methods may be of significant importance. To address the aforementioned issues, we can also find solutions from the deep learning wave triggered by AlexNet [63]. Compared to traditional machine learning algorithms, deep learning algorithms are neural network algorithms with deep structures within machine learning, boasting high performance, efficiency, and scalability. They can automatically extract image features, capture spatial relationships, and enhance detection of disease areas, thereby mitigating the impact of subjectivity in traditional machine learning processes and improving work efficiency. Deep learning algorithms have demonstrated powerful capabilities in complex nonlinear modeling tasks. Currently, many scholars have combined spectral imagery with deep learning methods for crop disease identification [28,64,65,66,67,68]. They primarily use spectral information and resolution to create datasets, and then incorporate new designs into existing networks to enhance the model’s extraction and representation capabilities. These datasets are then used to train models, enabling effective identification of crop diseases. However, these methods also have limitations. They are typically supervised learning methods, meaning that large-scale spectral datasets are required. The difficulties and limitations associated with collecting and labeling large-scale datasets cannot be ignored. Generating unlabeled spectral data using methods such as generative adversarial networks and diffusion models seems feasible, and we will discuss these methods in Section 6.

#### 3.2.2. Limitations of Datasets and Single Spectral Modality

Datasets are crucial in ensuring the reliability of artificial intelligence model training. However, there currently needs to be a unified and standardized spectral dataset for common crop diseases, which leads researchers to lack a comparison standard when building datasets or conducting experiments. This, in turn, makes it difficult to determine the reliability of the dataset and the model [62]. Therefore, one of the urgent tasks is to create a standard and open spectral disease dataset for crop diseases as much as possible. In addition, as shown in Table 4, researchers have selected different spectral bands for crop disease detection based on different crops. However, differences in spectral collection equipment in terms of resolution, bands, and wavelength range can lead to some loss and inadequacy in the crop disease features contained in the collected spectral images, posing challenges for the model to learn various disease features (Wei et al., Bu et al.). We can utilize the fusion of multiple image formats to complement each other and enhance the disease information contained in the images. Currently, many technologies for hyperspectral and multispectral image fusion, as well as the fusion of spectral images and RGB images, have been applied in the agricultural field [69,70,71,72] and have achieved excellent results. Therefore, selecting appropriate fusion algorithms to fuse multiple crop disease images to enhance crop disease features and improve the accuracy of crop detection is a noteworthy research area.

#### 3.2.3. Limitations of Experimental Analysis and External Influences

Currently, most disease detection methods based on spectral images do not focus on how spectral images reflect the changes in physicochemical indicators within infected crops. Instead, they primarily explore the feasibility of the established datasets on the applied models. This approach largely overlooks the ability of spectral images to reflect crops’ biophysical and biochemical characteristics. More analysis of this characteristic needs to be performed to prevent the establishment of a connection between the rich features of spectral images and the internal physicochemical changes in crop diseases. Consequently, researchers may need help to extract the most suitable feature information for the model, leading to misjudgments about model performance. Additionally, agricultural research has inherent delays, and images captured in specific time scenarios may be affected by external environmental changes such as leaf occlusion and brightness variations, impacting the reproducibility and validity of experiments [13]. Therefore, when collecting images, it is essential to comprehensively consider various potential factors and reduce the impact of external factors on the model, thereby achieving precise detection of crop diseases in the field.

## 4. Disease Recognition Methods Based on Red–Green–Blue Images

Compared to the difficulties and high costs associated with acquiring spectral images in field conditions, RGB images, characterized by their low cost, ease of acquisition, and simplicity of use [73], have become the more commonly used images in the application of computer vision technology in the agricultural field. RGB images, which simulate human vision in the visible spectrum, are more realistic color images that can more intuitively reflect disease areas and characteristics. As early as 2001, Takakura et al. [74] utilized RGB images for non-destructive testing of plant health. Although RGB images provide only limited information from three bands compared to spectral images, there have been multiple studies on crop disease identification based on RGB images. Regarding the limitations of spectral image-based disease identification technology discussed above, we mentioned the dependence of deep learning methods on large-scale datasets. However, acquiring RGB images is simpler and more convenient compared to spectral images, and the difficulty of constructing large-scale disease datasets is not high. Therefore, deep learning methods are more prevalent in research on crop disease detection based on RGB images.

It should be noted that traditional machine learning methods based on hand-crafted features, such as color, texture, and shape descriptors combined with classifiers (e.g., SVM and random forest), have also been explored in early RGB image-based disease recognition studies. However, due to their limited representation capacity and relatively weak generalization ability in complex field scenarios, such methods have become less dominant compared with deep learning approaches in recent studies.

### 4.1. Deep Learning Methods for Crop Disease Recognition Based on Red–Green–Blue Images

Deep learning is a branch of machine learning and a frontier area of artificial intelligence. It leverages multi-layered structures to represent abstract representations of data, thereby constructing computational models that can be iteratively trained. During the rapid development and widespread application era since the 2010s, deep learning technology has achieved remarkable progress and has been extensively applied in various fields. In 2012, deep learning’s outstanding performance in the ImageNet image recognition competition marked the maturity of its technology and the breadth of its applications. Subsequently, deep learning demonstrated powerful capabilities in multiple fields, such as face recognition, image generation, natural language processing, medical diagnosis, financial analysis, artistic creation, and autonomous driving. The success of these applications is not only attributed to the continuous optimization and innovation of deep learning algorithms but also benefits from the rapid development of technologies such as big data, high-performance computing, and cloud computing. With the continuous advancement of technology and increasingly diverse application scenarios, deep learning has become a significant force driving the development of artificial intelligence. It continues to bring revolutionary changes to human society.

**Figure 5:**
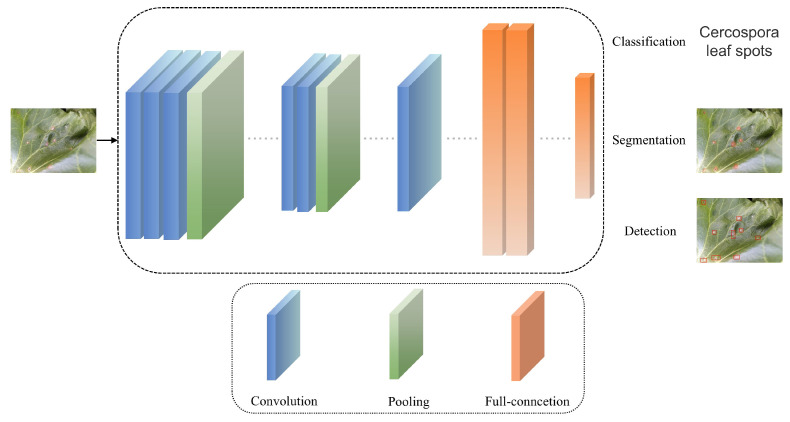
The basic process of RGB image-based deep learning crop disease recognition methods.

Disease recognition methods based on deep learning primarily leverage deep learning’s classification, segmentation, and object detection techniques. Figure 5 demonstrates the basic workflow of research on classification, segmentation, and object detection methods based on RGB images. These methods aim to identify and classify crop diseases accurately, primarily by training models on the disease regions of crop leaves in RGB images to recognize disease patterns, assisting people in determining the occurrence of diseases. Disease segmentation methods based on deep learning aim to segment the lesion areas from the leaves, accurately assigning each pixel in the lesion area to a predefined category, achieving pixel-level semantic understanding, and better-helping people understand the location, shape, and other information of the crop’s diseased areas. Disease detection methods based on deep learning can help people identify the location and disease category of crops, usually with high localization accuracy and classification accuracy.

Based on the selection criteria outlined in this paper, 45 studies from 2020 to 2025 on deep learning-based disease identification methods using RGB images were selected, Table 5 shows papers on crop disease recognition methods based on RGB images from 2020 to 2025, including their publication time, references, detection objects, baseline, improvement methods, and result.

### 4.2. The Limitations of Red–Green–Blue Image-Based Deep Learning Crop Disease Recognition Methods

Table 5 shows that most studies are conducted based on object detection methods. This is because classification and segmentation methods have certain limitations compared to object detection algorithms. For instance, classification methods typically accept inputs of narrow-scope images that contain only one or a few objects centered in the image, and they have limitations in classifying wide-scope images that contain multiple objects or even tens of objects [123]. Furthermore, these studies often emphasize the distinction between various disease types but cannot fully meet the need for flexible and real-time detection of specific disease locations [8]. Although segmentation methods, like detection methods, allow people to see the categories and locations of diseased leaves intuitively and can even reflect the shape of disease spots, segmentation methods have issues with difficulties in labeling and annotation [124]. Besides the problems caused by methodological limitations, deep learning-based disease identification methods using RGB images also face the following limitations and challenges.

#### 4.2.1. Lack of Crop Disease Data

**Figure 6:**
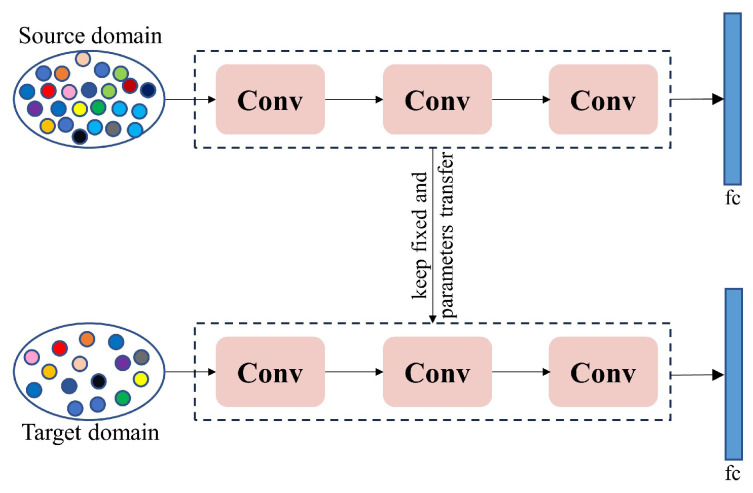
The basic process of transfer learning.

Although the acquisition and use of RGB images are cost-effective, the amount of data collected may sometimes fail to meet the requirements of deep learning models. Researchers and practitioners face significant data collection, labeling, quality assurance, and cost control challenges. Data augmentation techniques, which increase the quantity and diversity of data through operations such as translation, rotation, and cropping based on existing technology without additional labeling, are a commonly used improvement strategy [125]. Currently, researchers have combined multiple data augmentation strategies to enhance the diversity and complexity of models, improve their robustness, and prevent overfitting [75,85,96,101]. However, over-reliance on data augmentation can also lead to overfitting and may introduce new noise into the dataset. Alternatively, transfer learning (as shown in Figure 6) can also address the issue of poor model performance due to insufficient datasets [126]. When disease data is scarce, training on a larger dataset and then applying transfer learning to the crop diseases that need to be identified [127,128,129,130,131] is a practical approach. However, transfer learning also faces challenges posed by differences in dataset distributions. If the data distributions between the source and target data domains are significantly different, it will affect the effectiveness of model training [132]. These issues can be overcome through feature alignment, multitask learning, feature selection, or transformation. Additionally, we can utilize data synthesis techniques to generate diverse training samples by creating any number of synthetic data samples from simulated scenarios and then using them to train models [110,132].

#### 4.2.2. Lack of Publicly Available Standard Field Datasets

Although some public datasets on crop diseases have been proposed in recent years, most of them are laboratory-based rather than field-based. Consequently, models trained on these datasets may perform poorly in field conditions. Currently, disease recognition models developed by researchers are either based on partially public datasets or entirely on private datasets, and most of these datasets only contain a single crop species. Furthermore, researchers need to make these data public. Therefore, even for the same disease on the same crop, there is no unified standard to compare the performance of models developed by different researchers. Hence, images of common crop diseases must be captured in actual field conditions to construct benchmark datasets, providing research standards for relevant researchers.

#### 4.2.3. The Problems of Interference from Complex Scenes and Difficulty in Identifying Small Lesion Sizes

**Figure 7:**
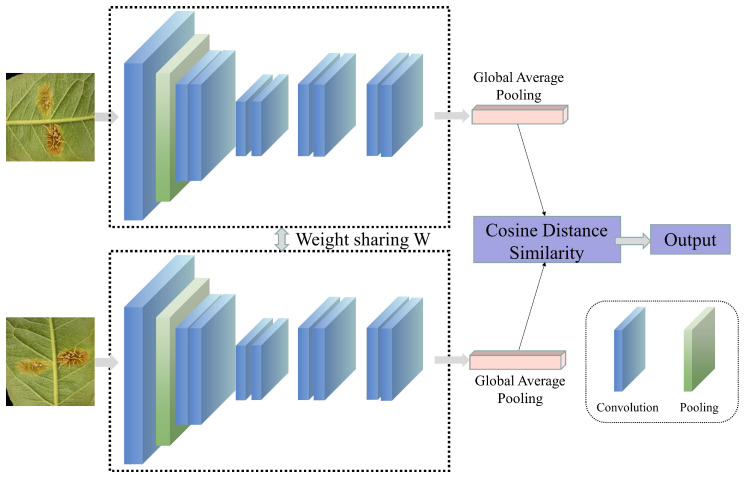
The basic process of contrastive learning.

In actual field scenarios, factors such as background interference, leaf occlusion, morphological differences, and scale variations may limit the model’s ability to extract disease features, thereby restricting the accuracy of disease recognition [133]. Furthermore, changes in weather and lighting conditions may introduce additional redundant features, affecting the model’s learning of disease-leaf features. When using drones with cameras for crop disease recognition, such dynamic background changes may make disease recognition even more difficult [14]. Additionally, the infection time and disease severity may vary among individual leaves in field scenarios. During the early stages of infection, when symptoms are relatively small, the difficulty of the model in recognizing the disease increases. There are several possible improvements to address the issues above. Firstly, complex example mining can be employed, which uses misdetected samples as new training samples to retrain the model, thereby enhancing its ability to recognize “hard-to-detect” samples [134]. This strategy has been widely applied in fields such as person recognition, speech recognition, disease recognition, and other agricultural areas [135,136,137,138,139,140,141]. Secondly, contrastive learning (as shown in Figure 7) can learn data representations by comparing the similarities and differences between different samples [142], enhancing the accuracy and generalization ability of crop disease recognition. Currently, commonly used contrastive learning methods in the field of crop disease recognition include image-pair contrastive learning [143,144,145,146] and self-supervised contrastive learning [147,148,149]. Additionally, researchers have addressed the issues of complex background interference and small lesion sizes making target recognition difficult to a certain extent by improving loss functions [67,88,96,117]. Alternatively, the attention mechanism in data can be leveraged to improve the model’s ability to focus on interfered areas and leaves with small lesion sizes, thereby enhancing recognition accuracy [75,96,98,106,150,151]. Building upon earlier CNN-based approaches, recent studies in 2024 and 2025 have increasingly adopted advanced Transformer-based and hybrid architectures to cope with complex field environments. Salman et al. [152] demonstrated that Vision Transformer-based models are particularly effective in in-the-wild scenarios by capturing long-range contextual dependencies, thereby improving the discrimination of disease symptoms from cluttered backgrounds. To further enhance robustness under varying illumination and background conditions, hybrid CNN–ViT frameworks have been proposed, combining local feature extraction with global contextual modeling [153]. In parallel, model interpretability has gained growing attention; explainable ensemble learning frameworks have been integrated to visualize the decision-making process, enhancing user trust and facilitating the distinction between true lesions and background noise [154].

## 5. Discussion

From a critical summary of existing research, we can understand that both spectral image-based and RGB image-based methods for crop disease recognition have achieved accuracy and reliability. However, we believe it is worth discussing how researchers should choose between spectral image-based and RGB image-based methods for crop disease recognition based on their own needs, as well as whether new models need to be developed for recognizing diseases in different crops or diseases within the same crop. In addition, it is necessary to discuss the applicability of traditional machine learning and deep learning methods under different image modalities and application scenarios.

Regarding the first question of whether to choose spectral or RGB images for crop disease recognition in one’s research, the essence of this issue lies in which stage of crop disease occurrence the research is targeting. If the aim is to utilize artificial intelligence technology before crop diseases spread on a large scale, then undoubtedly, spectral images are a better choice than RGB images. In such early detection scenarios, traditional machine learning methods combined with spectral features and physically interpretable indices can still be effective, while deep learning methods are increasingly adopted when sufficient labeled data are available. This is because before diseases spread widely, many crop leaves or fruits may appear visually indistinguishable from their healthy state, either to the human eye or in photos captured by RGB devices. However, they may already be affected internally by diseases, resulting in changes invisible to the human eye. Based on the characteristics of spectral images, spectral changes in crops that appear healthy on the surface but are infected can be detected before diseases spread widely, allowing for the identification of infected crops and the implementation of early measures to suppress the disease. However, experimental conditions are limited in actual research, such as the need for spectral capture equipment or only to identify the specific disease affecting crops once it has occurred. In that case, using RGB images is a better option. RGB images are more accessible and can be collected in the field using common smartphones. Furthermore, when a disease occurs, there are often prominent disease characteristics on the crop surface, making RGB images sufficient to meet recognition needs. For RGB images, deep learning methods dominate current research due to their strong feature learning capability, whereas traditional machine learning methods are more commonly reported in controlled environments or small-scale datasets. In summary, if conditions permit and early detection of crop diseases is required, then choosing spectral images for research is preferable; otherwise, using RGB images for research is sufficient.

Regarding the second question, researchers have developed different models for different diseases in different crops, for different diseases in the same crop, and even for the same disease in the same crop. This phenomenon needs to be viewed critically. For the first scenario, the leaves of different crops vary in size, shape, and other characteristics, and the diseases to be identified also differ. These significant differences may affect the ability of existing models to recognize unknown crop diseases. For the second scenario, significant differences in disease manifestations may also affect the ability to recognize existing models for crop diseases. However, if the differences in disease characteristics are insignificant, developing a new model may not be necessary. In addition, for the first two scenarios, incremental learning can help the model gradually absorb information from new data without forgetting old knowledge, thereby enabling effective recognition of both new and old data [155]. For the third scenario, perhaps developing an excellent method is sufficient, but scenario changes may lead to a decline in the model’s recognition performance for diseases. To address this issue, scene adaptation technology is a good solution [156]. This technology can help the model improve its recognition ability for the same category of targets in different scenarios.

## 6. Summary and Prospects

Crop diseases have caused irretrievable economic losses to the global agricultural industry. In contrast, traditional disease detection methods require a considerable workforce and material resources, and traditional disease control methods may cause environmental pollution and other issues. Therefore, there is a need for automated, economical, reliable, accurate, and rapid diagnostic systems to detect crop diseases. With the rapid development of artificial intelligence technology, an increasing number of researchers have applied AI methods to the field of disease recognition and achieved promising results. Many studies on crop disease recognition primarily utilize RGB images, multispectral images, and hyperspectral images. Therefore, this review provides a detailed overview of some publicly available datasets based on RGB and spectral images and some primary research methods. These methods are non-destructive and hold significant importance in the detection and prevention of agricultural crop diseases. It also summarizes some of the shortcomings of these methods and potential improvements. However, research on crop disease detection is still in a continuous development stage. Here, we discuss possible future trends and research directions in this field.

### 6.1. Integrating Multi-Source Data for Crop Disease Identification

The work summarized in this article shows that identification research efforts have relied solely on a single modality for recognition. However, single-modality approaches always have limitations in specific scenarios. Therefore, combining images from two modalities can enhance the efficiency of disease identification. There are primarily two ways to integrate spectral images with RGB images for crop disease identification: space–ground integration and multimodal fusion.

#### 6.1.1. Space–Ground Integration

The primary approach of space–ground integration involves using drones with spectral imaging devices to capture wide-range images during field patrols from high altitudes. Subsequently, disease recognition methods are employed to determine which farmland is affected by diseases, helping farmers pinpoint the locations of disease outbreaks. Once the locations are identified, farmers can visit the affected areas and capture RGB images using smartphones to obtain more detailed information about the diseases. Establishing and developing an integrated space–ground platform is a challenging but practical technical method. This approach ensures full-growth-cycle disease recognition for crops in actual field scenarios. Further, it enhances the accuracy of assessing the overall disease severity in farmland and identifying individual plant disease conditions.

#### 6.1.2. Multimodal Fusion

Multimodal fusion refers to integrating data from two or more modalities for crop disease identification, thereby overcoming the limitations of single-modality approaches. Its potential has been demonstrated in crop disease recognition [157,158]. By utilizing multimodal fusion techniques, such as extracting features from spectral and RGB images separately and then using a multi-level fusion model, the model’s performance can be enhanced to varying degrees. This is because the rich information in spectral images can compensate for the insufficient information obtained from RGB images when dealing with crops with mild diseases. Furthermore, RGB images can further supplement the information lacking in spectral images within the visible light spectrum. Combining multiple modalities allows the model to learn more comprehensive and sufficient phenotypic information, leading to superior performance. However, currently, most methods for multi-source data fusion focus more on the feasibility of this approach in downstream tasks. The key to the success of such research methods lies in how to efficiently and accurately align and fuse data from different modalities, which is also one of the potential research directions for the future.

### 6.2. Constructing a High-Quality Dataset

Although most current spectral disease identification methods are based on machine learning, deep learning represents the primary research direction for the future. Therefore, constructing high-quality datasets remains an inevitable challenge. While methods such as data augmentation and transfer learning have, to some extent, met the needs of disease identification, with the advancement of artificial intelligence technology, an increasing number of new data generation methods are worth exploring in the field of disease identification. Generative Adversarial Networks (GANs) may be a good choice, as they can be used for super-resolution reconstruction of images, thereby improving the quality of collected data and potentially enhancing model performance [159,160]. Additionally, GANs can generate images of crop diseases, enriching the dataset of crop disease images. In recent years, the rapid development of diffusion models cannot be ignored [161,162]. The generation of data using diffusion models has already been implemented in the agricultural field [163,164]. Therefore, utilizing diffusion models to generate crop disease data is also feasible.

### 6.3. Conduct Research on Disease Identification Using Video Modality

Instead of having farmers conduct field inspections in person or using drones for field patrols for disease identification, installing cameras in the field for round-the-clock, all-weather disease identification may be a superior approach. Research on video-based disease identification methods is indispensable in utilizing cameras for disease identification. Furthermore, this may also lead to the development of rain and fog removal technologies, thereby enhancing the universal applicability of camera-based disease identification. Looking beyond specific sensing modalities, recent studies indicate a clear trend toward large-scale pre-trained and cross-modal learning frameworks for intelligent crop disease diagnosis. Recent reviews highlight that integrating deep representation learning with broader contextual information can significantly improve generalization performance under limited labeled data [27,165]. In addition, emerging cross-modal architectures, such as hybrid CNN–GNN models with attention mechanisms, demonstrate the potential of combining visual perception with structured agronomic knowledge for more interpretable disease diagnosis [166]. Together with advances in explainable and human-centered AI, these developments point toward more interactive and knowledge-aware crop protection systems in future precision agriculture.

## Figures and Tables

**Table 1:** Comparison between existing reviews and this review in the field of crop disease detection.

Work	Citation	Focus
[9]	445	Introduced an investigation on methods for detection and classification of leaf diseases in citrus plants.
[11]	82	Convolutional neural networks (CNNs) for diseases in vegetables, fruits, and miscellaneous plants
[12]	220	The current status of using Convolutional Neural Networks (CNNs) for identifying and diagnosing plant diseases.
[13]	136	Introduced the modern advancements in early plant disease detection based on hyperspectral remote sensing.
[14]	124	Reviewed the application of vision-based machine learning techniques in plant disease detection.
[15]	32	Reviewed the application of machine learning and deep learning methods in the identification of diseases in four crops: tomatoes, peppers, potatoes, and cucumbers.
[10]	200	Provided a comprehensive review of deep learning approaches for plant disease detection, with particular emphasis on Generative AI, Foundation Models, and emerging real-time detection trends up to 2025.
Present review	-	Reviewed studies on crop disease identification based on spectral images and RGB images.

**Table 2:** Publicly available datasets. Abbreviations: RGB = red–green–blue images; Hyperspectral = hyperspectral images with tens to hundreds of continuous spectral bands; XDB = Extended Dataset for Plant Disease Detection; SWD = Strawberry Wilt Disease dataset; NLB = Northern Leaf Blight dataset; FGVC8 = Fine-Grained Visual Categorization 8 (Plant Pathology); LWDCD2020 = Leaf Wilt Crop Disease Dataset 2020; AFB = American Foulbrood dataset.

Dataset	Classes	Image Type	Sizes	Numbers	URL (Accessed on 20 December 2025)
PlantVillage [16]	38	RGB	256 × 256	54,305	https://data.mendeley.com/datasets/tywbtsjrjv/1
XDB [17]	171	RGB	4128 × 3096 3096 × 4128	46,513	https://www.digipathos-rep.cnptia.embrapa.br/
SWD [18]	4	RGB	1024 × 768	3531	https://github.com/boxyao/Strawberry_wilt_dataset
NLB [19]	1	RGB	3353 × 2235 5919 × 3952 6000 × 4000	18,222	https://osf.io/p67rz/
FGVC8 [20]	12	RGB	4000 × 2672	18,632	https://www.kaggle.com/c/plant-pathology-2021-fgvc8
LWDCD2020 [21]	4	RGB	Unfixed size	12,000	https://drive.google.com/drive/folders/1XlwoD8xes1punAzlGSz8mluQXm-CXcOJ
Plantdoc [22]	17	RGB	Unfixed size	2598	https://github.com/pratikkayal/PlantDoc-Dataset
BARI-Sunflower [23]	3	RGB	512 × 512	467	https://data.mendeley.com/datasets/b83hmrzth8/1
Katra-Twelve [24]	12	RGB	6000 × 4000	4503	https://data.mendeley.com/datasets/hb74ynkjcn
AFB [25]	1	Hyperspectral	512 × 512	346	https://entrepot.recherche.data.gouv.fr/dataset.xhtml?persistentId=https://doi.org/10.57745/R6AMN3

**Table 3:** Commonly used evaluation metrics in crop disease recognition.

Metric	Formula	Description
Accuracy	$ACC=\frac{TP+TN}{TP+TN+FP+FN}$	Overall correctness of classification.
MCC	$MCC=\frac{TP\times TN-FP\times FN}{\sqrt{\left(TP+FP)(TP+FN)(TN+FP)(TN+FN\right)}}$	MCC reflects the correlation between true disease categories and predicted disease categories by calculating their correlation coefficient; the higher the correlation, the better the prediction performance.
Kappa	$Kappa=\frac{P_{0}-P_{e}}{1-P_{e}}$	Measures inter-rater agreement adjusted for chance.
Precision	$P=\frac{TP}{TP+FP}$	Proportion of predicted positives that are actually positive.
Recall	$R=\frac{TP}{TP+FN}$	Proportion of actual positives correctly identified by the model.
F1 Score	$F1=\frac{2\times P\times R}{P+R}$	Harmonic mean of precision and recall.
AP	$AP=\int_{0}^{1}P\left(R\right)\;dR$	Area under the precision–recall curve; measures detection performance for one class.
mAP	$mAP=\frac{1}{N}\sum_{i=1}^{N}AP_{i}$	Mean of AP across all classes; evaluates overall detection performance.
RMSE	$RMSE=\sqrt{\frac{1}{m}\sum_{i=1}^{m}{\left(y_{i}-t_{i}\right)}^{2}}$	Root mean squared error; measures prediction deviation from actual values.
$R^{2}$	$R^{2}=1-\frac{\sum_{i=1}^{m}{\left(y_{i}-t_{i}\right)}^{2}}{\sum_{i=1}^{m}{\left(y_{i}-\bar{y}\right)}^{2}}$	Indicates proportion of variance in dependent variable explained by the model.
MAE	$MAE=\frac{1}{m}\sum_{i=1}^{m}\left|y_{i}-t_{i}\right|$	Mean absolute error; average of absolute prediction errors.

Note: TP, TN, FP, and FN denote true positives, true negatives, false positives, and false negatives, respectively. $P_{0}$ and $P_{e}$ represent the observed agreement and the expected agreement by chance in Cohen’s Kappa. *P* and *R* denote precision and recall, respectively. $AP_{i}$ is the average precision for the *i*-th class, and *N* is the total number of classes. $y_{i}$ and $t_{i}$ denote the predicted value and the ground-truth value of the *i*-th sample, $\bar{y}$ is the mean of ground-truth values, and *m* is the total number of samples.

**Table 4:** Spectral image-based traditional machine learning crop disease recognition methods.

Reference	Crop	Disease	Camera Model	Wavelength (nm)	ML	Best Results
[35]	Cotton	Wilt	MCA Snap Zenmuse X5R	Near infrared1 80 ± 10 Blue 490 ± 10 Green 550 ± 10 Red 680 ± 10 Red edge 720 ± 10 Near infrared2 900 ± 2	**GWO-ELM** PSO-BP LR	The $R^{2}$ values for the flowering stage and the boll stage are 0.65 and 0.88, respectively.
[36]	Wheat	Crown rot	FX10 SWIR	400–1000	**SVM**	F1-score is above 0.75.
[37]	Wheat	Fusarium head blight	GaiaField-V10E	361–1011	**XGBoost** KNN RF SVM NN	XGBoost achieved average accuracies of 95.46%, 91.36%, and 96.74% on WFSI1, WFSI2, and the combination of WFSI1 and WFSI2, respectively.
[38]	Cotton	Wilt	SR-3500	350–2500	**LR** SVM KNN	The average accuracy is 90.62%.
[39]	Wheat	Fusarium head blight	ASD FieldSpec 4 Hi-Res Malvern Panalytical	350–2500	**KNN** RF SVM	$R^{2}$ and RMSE are 0.92 and 10.21, respectively.
[40]	Wheat	Stripe rust	ASD FieldSpec QE Pro	350–2500 644–806	**XGBoost** PLSR BPNN RF	$R^{2}$ and RMSE are 0.867 and 0.104, respectively.
[41]	Potato	Late blight	CMOS	Blue 450 ± 16 Green 560 ± 16 Red 650 Red edge 730 ± 16 Near infrared 840 ± 26	**RF** SVM KNN	The overall accuracy and Kappa are 97.50 and 0.96, respectively.
[42]	Citrus	Anthracnose	GaiaField-V10E	388–1025	**SVM** KNN RF	The average accuracy is 91.04%.
[43]	Wheat	Stripe rust	QE Pro	644–806	**GPR**	The $R^{2}$, RMSE, and MAE are 0.912, 0.097, and 0.071, respectively.
[44]	Banana	Wilt	RedEdge MX Zenmuse X7	Blue 475 ± 10 Green 560 ± 10 Red 668 ± 5 Red edge 717 ± 5 Near infrared 840 ± 20	**RF** SVM BPNN	The overall accuracy is 97.28%.
[45]	Wheat	Fusarium head blight	FX10	400–1000	**SVM** NN LR	The classification accuracy is 95.6%.
[46]	Wheat	Stripe rust	ASD FieldSpec 4	350–2500	**XGBoost** GBRT	The $R^{2}$ and RMSE are 0.8894 and 0.1135, respectively.
[47]	Apple	Fire blight	MicaSense RedEdge-M	Blue 475 ± 20 Green 560 ± 20 Red 668 ± 10 Red edge 717 ± 10 Near infrared 840 ± 40	**RF** DT SVM	The overall accuracy and Kappa are 94.0% and 0.904, respectively.
[48]	Wheat	Fusarium head blight	ASD FieldSpec 4	350–2500	**RF** KNN SVM NN XGBoost	When the severity of the disease is 10.78%, the classification accuracy is 100%.
[49]	Wheat	Powdery mildew	GaiaField-V10E	400–1000	**PLSR**	The classification accuracy is 91.4%.
[50]	Sugarcane	White leaf	P4 Mulitspectral	Blue 450 ± 16 Green 560 ± 16 Red 650 ± 16 Red edge 730 ± 16 Near infrared 840 ± 26	**XGBoost** KNN RF DT	The overall accuracy is 94%.
[51]	Grape	Esca disease	Xeva 1.7-320 InGaAs	900–1700	**PLS-DA**	The accuracy is in the range of 82.78–95.53%.
[52]	Rice	Blast disease	FieldSpec 4 Hi-Res	350–2500	**SVM** KNN LDA	The classification accuracy of asymptomatic, early infection stage and mild infection stage are 65%, 80%, and 95%,
[53]	Wheat	Powdery mildew	GaiaField-V10E	400–1000	**PLSR**	The overall accuracy and Kappa are 82.35% and 0.56, respectively.
[54]	Potato	Late bligh	MicaSense RedEdge	Blue 475 ± 20 Green 560 ± 20 Red 668 ± 10 Red edge 717 ± 10 Near infrared 840 ± 40	**LSVC** RF SVM KNN	The average overall accuracy and average are 0.837 and 0.622, respectively.
[55]	Wheat	Fusarium head blight	Cubert S185 FireflEYE SE	459–950	**LR**	The accuracy, F1-score, and AUC-ROC are 0.90, 0.83, and 0.82, respectively.
[56]	Grape	Leafroll disease	Micro-Hyperspec NIR X-Series	517–1729	**LS-SVM**	The classification accuracy is in the range of 66.67–89.93%.
[57]	Cotton	Root rot	CMOS AF Nikkor	-	**LR**	The classification accuracies of the spectral model, texture model, and spectral-texture model are 92.95%, 84.81%, and 91.87%, respectively.
[58]	Mango	Anthracnose	Spectral Evolution SM-1900	350–1900	SVM RF	The 29 significant bands identified through LDA outperform the bands obtained other methods in terms of performance for SVM and RF.
[59]	Citrus	Greening	ADC-lite	Green 520–600 Red 630–690 Near infrared 760–900	**AdaBoost** XGBoost RF SVM KNN LR NB	AdaBoost using a threshold strategy achieved 100% accuracy.
[60]	Banana	Wilt	MicaSense RedEdge MTM	Blue 465–485 Green 550–570 Red 653–673 Red edge 712–722 Near infrared 800–880	**LR**	The average overall accuracy and average Kappa are 85.85% and 0.71, respectively.
[61]	Potato	Early blight	Imspector V9	430–900	**SVM** PLS-DA	The accuracy is 0.92.
[30]	Wheat	Stripe rust	Cubert S185	450–950	**Hybrid DL (3D-CNN based)**	Deep spectral–spatial features achieved superior performance compared with traditional machine learning models.
[31]	Apple	Quarantine diseases	Hyperspectral camera	Full spectral range	**3D-CNN**	The proposed model achieved high accuracy in early disease detection.
[32]	Wheat	Multiple infections	Hyperspectral system	VIS–NIR	**Deep learning**	The method effectively distinguished overlapping disease symptoms.

**Table 5:** Red–green–blue image-based deep learning crop disease recognition methods.

Reference	Crop	Disease	Baseline	Improvements	Results
[75]	Wheat	Fusarium head blight	YOLOv5	Integrated a parallel channel-spatial attention module into the feature fusion module.	Accuracy, recall and mAP are 80.6%, 74.5% and 83.2%, respectively.
[76]	Cauliflower	Bacterial Soft Rot Downy Mildew Black Rot	YOLOv8	Added an additional Conv block in the head section. Replaced the original activation function with Hard Swish.	Accuracy, recall and mAP are 93.2%, 82.6% and 91.1%, respectively
[77]	Grape	Powdery mildew Anthracnose Gray moid White rot	YOLOv5	Integrated a VoVNet-based GC-RE-OSA module in the backbone. Used an improved real-time Transformer in the neck. Added 2D positional embeddings and SSTE to the final feature map.	Parameters, mAP, and FPS are 24.5 MB, 90.67%, and 44, respectively.
[8]	Wheat	Fusarium head blight	YOLOv5	Used MobileNetV3 as the backbone. Replaced a portion of the feature fusion module’s C3 module with C3Ghost.	The mAP is 97.15%, the FLOPS increases by 71.32% compared to YOLOv5.
[78]	Apple Pom Mango Jamun	Rust Alternaria blotch Gray spot Cercospora spot Anthracnose Fungal disease	Mask R-CNN	Designed Dual Scale Overlap (DSO) patch embedding to effectively extract multi-scale disease features through dual paths, reducing the omission of lesions. Customized an Ultra Large Convolution (ULC) Transformer block for positional encoding and global modeling, efficiently extracting global and positional features of leaves and diseases. Proposed the Skip Convolutional Local Optimization (SCLO) module to optimize local details and edge information, enhancing the model’s pixel classification ability, resulting in finer segmentation of leaves and spots, and enabling the extraction of smaller lesions. Built a Double Layer Upsampling (DLU) decoder to efficiently fuse detailed information with semantic information and output accurate segmentation results of leaves and spots.	The IoU of lesion segmentation achieve 94.47%, 94.54%, 83.83%, 86.60%, 89.59% and 88.76%, respectively.
[75]	Wheat	Fusarium head blight	YOLOv5	Integrated a parallel channel-spatial attention module into the feature fusion module.	Accuracy, recall and mAP are 80.6%, 74.5% and 83.2%, respectively.
[79]	Wheat	Stripe rust Powdery mildew Scab	Mask R-CNN	Used Densely Connected Convolutional Networks (DenseNet) for preliminary feature extraction. Designed a backbone feature extraction network combining Feature Pyramid Network (FPN) and attention mechanism to extract diversification-augmented features. Developed an Edge Agreement Head module based on Sobel filters to compare edge features during training, thereby improving the model’s mask generation efficiency.	The mAP is 96.02%
[80]	Grape	Black rot Esca black measles Leaf blight Powdery mildew	VGG-19	The training set for the recognition model is pre-extracted using grayscale technology and grid-point positioning methods, allowing for the early formation of filters and the construction of convolution kernels.	The parameters of the improved VGG-19 were reduced to 38.5 MB, with a training time of only 49 min and 41 s, and an accuracy rate of 96.25% on the validation set.
[81]	Apple	Healthy Spotted leaf disease Mosaic disease Leaf rust Yellowing Leaflet disease Leaf brown spot Glomerella leaf spot Powdery mildew	-	A new lightweight network, RepDI, was proposed, and it became CPU-friendly due to structural reparameterization. Channel and spatial compression, combined with dilated convolution, facilitated the extraction of global information, improving the model’s recognition ability in complex backgrounds.	Achieved the fastest inference speed on desktop CPUs and reached a Top-1 accuracy of 98.92%
[82]	Strawberry	Normal strawberry fruit Normal strawberry leaves Gray mould Gnomonia fructicola fall Fertilizer damage Blight Pestalotiopsis leaf spot Calcium deficiency Fargaria ananassa duch Common leaf spot Anthracnose High-temperature damage of strawberries	Vision transformer	Utilized the self-attention mechanism, Multi-Head Self-Attention (MSA) was employed to capture long-range feature dependencies in strawberry disease images. A Transformer based on Spatial Convolutional Self-Attention (SCSA-Transformer) was proposed to reduce the parameters of the Transformer network and enhance recognition efficiency.	Recognition accuracy is 99.10%.
[83]	Tomato	Healthy Mosaic virus Yellow leaf curl virus Early blight Late blight Leaf mold Septoria leaf spot Target leaf spot Bacterial leaf spot Spider mite damage	ResNet50	Without adding parameters or further boundary annotations, the addition of the target localization module enables reliable prediction of lesion positions. By erasing contextual semantic data, the proposed destruction and reassembly module is able to learn more granular features. The addition of the attention area division module can locate lesion positions, thereby improving recognition accuracy.	The accuracy rates for identifying the severity of tomato diseases and for recognizing the types of tomato diseases have reached 95.03% and 98.25% respectively.
[84]	Apple	Frog eye spot Powdery mildew Rust Scab	YOLOv5	Incorporated the BCM module into the backbone. Integrated cross-attention mechanism and bidirectional transposed feature pyramid network into the feature fusion module.	Accuracy and FPS are 85.23 and 33, respectively.
[85]	Tea	Leaf blight	YOLOv5	Added multi-scale RFB modules in the backbone. Added dual-dimensional mixed attention in the Neck section.	Accuracy, recall, F1-socre, AP@0.5, and AP are 72.2%, 71.1%, 71.6%, 76.8% and 44.5%, respectively.
[86]	Maize	Anthracnose leaf blight Tropical rust Southern maize rust Common rust Southern leaf blight Phaeosphaeria leaf spot Diplodia leaf streak Physoderma brown spot Northern leaf blight	Faster R-CNN	Added batch normalization layers in the convolutional layers. Proposed a central cost function to construct a hybrid loss function.	Average recall, accuracy, and F1-score are 0.9719, 97.23 and 0.9718, respectively.
[87]	Apple	Alternaria blotch Brown spot Grey spot Mosaic Rust	YOLOv5	Reconstructed the backbone using lightweight Shufflenet reverse residual blocks (SNIR). Introduced an efficient feature learning module (DWC3) with a depthwise separable convolution design in the neck part. Integrated lightweight coordinate attention modules (CA) into the Backbone and neck parts. Replaced CIOU with SIOU as the bounding box regression loss function.	The mAP and detection speed are 95.5% and 625 frames per second, respectively.
[88]	Grape Peach Potato Apple Corn	Black measles Leaf blight Block rot Bacterial spot Late blight Block rot Scab Northern leaf blight	YOLOv5	Improved the feature fusion module using lightweight structures and multi-branch structures. Proposed CAM to extract global and local features from each network layer. Added an additional grid to predict targets and modified the formula for the centroid offset of the prediction box. Used an improved DIoU loss function to replace GIoU.	The mAP, F1-score, and recall are 95.92%, 0.91, and 87.89%, respectively.
[89]	Tobacco	Brown spot	YOLOX	Introduced hierarchical mixed-scale units (HMUs) in the neck part. Introduced Convolutional Block Attention Module (CBAM) into the network.	Average accuracy is 80.56%.
[90]	Maize	Blight Leaf spot Mosaic	YOLOv8	-	Accuracy is 99.04%.
[91]	Tea	Leaf blight	YOLOv5	Integrated self-attention and convolution (ACmix) as well as CBAM into YOLOv5. Replaced the SPPF module with the RFB module. Introduced the Global Context Network (GCNet).	AP is 73.7%.
[92]	Strawberry	Angular leafspot Anthracnose Blossom blight Gray mold Leaf spot Powdery mildew	Faster R-CNN	Constructed a multi-scale feature fusion network using ResNet, FPN, and CBAM.	The mAP is 92.18%.
[93]	Grape	Black measles	DeepLabV3+	Used DeepLabV3+ to extract features of ROI and POI. Developed a fuzzy rule-based system for each feature to predict the severity of the disease. Considered appropriate membership functions for the inputs and outputs for the fuzzification and defuzzification processes.	The accuracy of the overall disease severity classification is 97.75%.
[94]	Tomato Cucumber Cabbage	Umbilical rot Gray mould Target spot Authracnose	YOLOv5	Embed the Transformer encoder into the CSP structure. Integrated the improved InceptionA module into the FPN structure. Improved NMS with the Confluence module.	The mAP is 93.1%.
[95]	Jute	Stem rot Anthracnose Black band Soft rot Tip blight Dieback Mosaic Chlorosis	YOLOv5	Utilized SCFEM, SPPM, and DSCFEM in the backbone. Collected cross-stage features from three different levels of the backbone in the Neck. Merged the anchor box results at different scales to create aggregated detection boxes.	The mAP and F1-score are 96.63% and 95.83%, respectively.
[96]	Cotton	Brown spot Red leaf blight Verticillium wilt	YOLOX	Introduced the ECA module into YOLOX. Used the Focal Loss loss function. Added the Hard-Swish activation function.	The mAP is 94.60%.
[97]	Citrus	Greening disease	YOLOv5	-	YOLOv5l-HLB2’s F1-score is 85.19%.
[98]	Tomato	Virus	YOLOv5	Combined the SE module with each Res unit of the backbone network to form SE-Res modules.	Accuracy and mAP@0.5 are 91.07% and 94.10%, respectively.
[99]	Apple	Rust Scab Blotch	YOLOX	Designed asymmetric ShuffleBlock, CSP-SA modules, and separable convolutions to improve the backbone network.	The mAP on MSALDD and PlantDoc are 91.08% and 58.85%, respectively.
[100]	Tomato	Bacterial spot Leaf mold Septoria spot Target spot Mosaic Early blight	Mask R-CNN	Added a lightweight head. Modified the aspect ratios of the RPN network’s anchor boxes and the feature extraction topology.	Accuracy, mAP, and F1-score are 0.98, 0.88, and 0.912, respectively.
[101]	Tomato	Early blight Late blight Leaf mold Septoria leaf spot	YOLOv4	Incorporated DenseNet into the backbone. Added two new residual blocks to both the backbone and the neck. Added CSP2-n modules to PANet. Used the Hard-Swish function as the primary activation function.	Precision, F1-score, and mAP are 90.33%, 93.64%, and 96.29, respectively.
[102]	Grape	Downy mildew	YOLOv5	Integrated Coordinate Attention (CA) into YOLOv5.	Precision, mAP@0.5, and recall are 85.59, 83.70%, and 89.55%
[103]	Cucumis melo	Powdery mildew	YOLOv5	Reconstructed the backbone of YOLOv5 using ShuffleNetv2 reverse residual blocks. Trimmed and fine-tuned the model by applying a channel pruning method.	The mAP@0.5 is 93.2%.
[104]	Strawberry	Powdery mildew	YOLOv4	Replaced the backbone and neck with versions that incorporate depthwise convolution and a hybrid attention mechanism.	The mAP is 72.7%
[105]	Cucumber	Powdery mildew Downy mildew Anthrax	YOLOv5	Integrated Coordinate Attention (CA) and Transformer. Combined multi-scale training strategy (MS) with a feature fusion network.	Model size, FLOPS, mAP, and FPS are 4.7 MB, 6.1G, 84.9%, and 143, respectively.
[106]	Maize	Healthy Gray leaf spot Common rust Northern leaf blight Fallarmy worm Herbicideburn Zincdeficiency	-	Used Hard Coordinated Attention (HCA) assigned at different spatial scales to extract features from maize leaf disease images, reducing the influence of complex backgrounds. Constructed a multi-feature fusion network that extracts weight information in two spatial directions, maximizing the retention of maize leaf disease features during the sampling process. Replaced the traditional convolutional layers with depthwise separable convolutional layers, reducing the number of parameters in the network.	The average recognition accuracy and F1 score were 97.75% and 97.03%, respectively.
[107]	Apple Tomato Potato Strawberry Chili	The severity of leaf disease	SSD	Se_SSD integrated the SSD feature extraction network with a channel attention mechanism. DB_SS improved the VGG feature extraction network. DBA_SSD integrated the improved VGG network with a channel attention mechanism.	DBA_SSD achieves the accuracy of 92.20%.
[108]	Cucumber	Downy mildew Bacterial angular spot	SSD	Selected EfficientNet-b1 as the backbone network. Selected the ASFF feature fusion method.	Model size, GFLOPS, and mAP are 7.72 MB, 0.14, and 85.52%.
[109]	Wheat	Health Glume blotch Scab	-	Designed a lightweight convolutional neural network (CNN) model called SimpleNet. Introduced the Convolutional Block Attention Module (CBAM) to enhance the model’s ability to represent disease features. Designed a feature fusion module to concatenate the down-sampled feature maps output by inverted residual blocks with the average pooled features of the input feature maps, achieving fusion between features of different depths.	The recognition accuracy on the test dataset reached 94.1%.
[110]	Soybean	Bacterial spot Virus disease Frogeye leaf spot	Faster R-CNN	Connected different layers in the feature extraction network in a skipping manner.	AP is 83.34%.
[111]	Apple	Alternaria blotch Brown spot Gray spot Rust Mosaic	SSD	Proposed a basic module called MEAN. Utilized GoogleNet’s Inception module. Replaced all convolutional kernels in Inception with MEAN modules.	The mAP and FPS are 83.12% and 12.53, respectively.
[112]	Grapevine	Leaf black rot	YOLOv3	Replaced the loss function with GIOU. Added SPP (Spatial Pyramid Pooling). Enhanced the training images through the BL super-resolution method.	The PlantVillage dataset’s accuracy and recall are 95.79% and 94.52%, respectively. The orchards dataset’s accuracy and recall are 86.69% and 82.27%, respectively. On the images without background clutter’s accuracy and recall are 94.05% and 93.26%.
[113]	Tomato	Early bight Gray mold Yellow leaf curl Brown spot Gray mold Leaf mold Navel rot Leaf curl Mosaic	YOLOv3	Optimized the feature layer of the YOLOv3 model using an image pyramid to achieve multi-scale feature detection.	The accuracy is 92.39%, and the detection time is only 20.39ms.
[114]	Wheat	Stripe rust	DenseNet	Embed the Convolutional Block Attention Module (CBAM) into the Densely Connected Convolutional Network (DenseNet).	The recognition accuracy on the test dataset reached 97.99%.
[115]	Tomato	Early blight Late blight Citrinitas leaf curl leaf mold Bacterial leaf spot	ResNet50	Denoised and enhanced the image by combining Wavelet Transform with Retinex (BWTR), removing noise points and edge points while retaining important texture information. Separated the tomato leaves from the background using the KSW method optimized by the Artificial Bee Colony algorithm (ABCK). Recognized the image using the Both-channel Residual Attention Network model (B-ARNet).	The overall detection accuracy is approximately 89%.
[116]	Grape	Black measles Black rot Leaf blight Mites of grape	Faster R-CNN	Introduced Inception-v1 modules, Inception-ResNet-v2 modules, and SE (Squeeze-and-Excitation) modules.	The mAP and FPS are 81.1% and 15.01.
[117]	Tomato	Gray leaf spot	YOLOv3	Used MobileNetv2 as the backbone network. Introduced the GIoU bounding box regression loss function.	F1-score, AP, and average IOU are 94.13%, 92.53%, and 89.92%.
[118]	Tomato	Leaf mold fungus Powdery mildew Blight Tomv	Faster R-CNN	Replaced VGG16 with a deep residual network. Utilized the K-means clustering algorithm to obtain anchor boxes.	The mAP is 97.18%
[119]	General	Leaf diseases	YOLOv8	Integrated the YOLOv10 architecture with an NMS-free training strategy to improve detection efficiency.	The mAP on the COCO dataset is 52.5%, with superior inference speed in terms of FPS.
[120]	Banana	Sigatoka leaf spot	YOLOv8	Designed a lightweight YOLOv10 detection head to enable efficient real-time disease detection.	The classification accuracy reached 98.5%, with an inference speed of 120 FPS.
[121]	General	Leaf diseases	Vision Transformer (ViT)	Proposed a Vision Transformer-based framework for leaf disease detection in precision agriculture, enhancing model generalization capability.	The proposed method achieved an accuracy of 97.8% and an F1-score of 0.96.
[122]	General	Leaf diseases	ResNet	Introduced VMamba, a visual state space model with linear computational complexity for efficient feature learning.	The model achieved an accuracy of 99.1% and demonstrated faster inference speed compared with DeiT.

## Data Availability

No new data were created or analyzed in this study. Data sharing is not applicable to this article.

## Nomenclature

		NN	Neural Network
GBRT	Gradient Boosting Regression Tree	LSVC	Linear Support Vector Classifier
NB	Naive Bayes	PLSR	Partial Least Squares Regression
GWO-ELM	Grey Wolf Optimization-Extreme Learning Machine	BPNN	Backpropagation Neural Network
PSO-BP	Particle Swarm Optimization-Backpropagation Neural Network	DT	Decision Trees
SVM	Support Vector Machine	PLS-DA	Partial Least Squares-Discriminant Analysis
KNN	K-nearest Neighbors	LDA	Linear Discriminant Analysis
RF	Random Forest	LS-SVM	Least Squares-Support Vector Machine
GPR	Gaussian Process Regression	AdaBoost	Adaptive Boosting
LR	Logistic Regression	XGBoost	Extreme Gradient Boosting
